# Impact of Albendazole on Cytokine and Chemokine Response Profiles in *Echinococcus multilocularis*-Inoculated Mice

**DOI:** 10.1155/2021/6628814

**Published:** 2021-05-07

**Authors:** Jing Wu, Hai-zhang Ma, Shadike Apaer, Nuerzatijiang Anweier, Qi Zeng, Xiafukati Fulati, Tao Li, Jin-ming Zhao, Hao Wen, Tuerhongjiang Tuxun

**Affiliations:** ^1^State Key Laboratory of Pathogenesis, Prevention, Treatment of High Incidence Diseases in Central Asia, Xinjiang Medical University, Urumqi 830054, China; ^2^Department of Liver & Laparoscopic Surgery, Digestive & Vascular Surgery Center, The First Affiliated Hospital of Xinjiang Medical University, Urumqi 830054, China; ^3^Department of Organ Transplant, Qilu Hospital of Shandong University, Jinan 250012, China

## Abstract

**Objective:**

Alveolar echinococcosis (AE) is a zoonosis caused by the larval stage of the metacestode *Echinococcosis multilocularis* with a tumor-like behavior in the targeted organ, especially in the liver. Surgery with albendazole is first-line modality for AE. Drug discontinuation is usually based upon the parasitic viability shown by the positron emission tomography (PET) scan. However, as a demanding and expensive method, it is not widely practiced in majority of the endemic regions. Further understanding on the cytokine and chemokine response profiles in AE patients may provide an interesting insight for potential markers in viability assessment.

**Methods:**

Mice were inoculated with *Echinococcus multilocularis* intrahepatically to develop the hepatic AE murine model. Oral albendazole administration was then applied for three months after the first inoculation, and peripheral and regional immune cells including type 1 T helper cells (Th), Th2, Th17, regulatory T (Treg) cells, related cytokines, and chemokines were examined.

**Results:**

The hepatic AE lesion was confirmed by ultrasound examination resulting in a successful rate of 70%. Among the 17 cytokines and chemokines detected, plasma levels of IL-23 were significantly higher in *E. multilocularis*-infected mice when compared to the control group; furthermore, more obvious increasing levels were found after albendazole treatment (*p* < 0.05). All chemokine levels other than eotaxin and MCP-3 were slightly higher in *E. multilocularis*-infected mice compared to the control group (*p* > 0.05). Eotaxin levels were significantly decreased in mice with *E. multilocularis* infection followed by albendazole treatment (*p* < 0.05). Both IL-17A and IL-23 expressions in hepatic AE lesions were significantly higher and related with disease activity.

**Conclusion:**

Albendazole administration influenced the balance of immune response and promotes the secretion of proinflammatory factors which is beneficial to parasite clearance. IL-23 seems to be associated with the successful albendazole treatment in mice with *E. multilocularis* infection; such a change could be translated into clinical application in the near future.

## 1. Introduction

Alveolar echinococcosis (AE), a globally distributed zoonotic disease, is caused by the larval stage of the *Echinococcus multilocularis* metacestode. China is one of the mostly affected countries and is a source of nearly 90% of the newly occurring AE patients [1]. Despite its relatively low incidence ranging from 0.03 to 1.2 per 100,000 [2], it is accompanied with a mortality of greater than 90% if inadequately treated. The parasite dwells mainly in the liver, growing through its infiltrative behavior, thus providing adjacent liver parenchymal damage, fibrosis, and even failure.

Radical surgery is the only curative option with long-term survival. Unfortunately, only a few AE patients benefited from radical resection due to the “silent” growth of the parasite and delayed diagnosis, treatment, and follow-up especially in poorly resourced areas. Benzimidazole as an antiparasitic chemical compound has greatly improved the clinical outcome of AE patients [3]. To date, albendazole (ABZ) is proved to be the most clinically efficient benzimidazole, being recommended as the preferred drug for interrupting larval growth by the World Health Organization Informal Working Group on Echinococcosis (WHO-IWGE) [4]. Currently, there are few effective means to predict the curative effect of albendazole. It is now well accepted that ^18^F-fluorodeoxyglucose positron emission tomography/computed tomography (^18^F-FDG PET/CT) is the only diagnostic method evaluating the metabolic activity of AE lesions [5, 6]. PET scan not only determines the boundary of the lesion but also assesses metabolic activity. It could provide clinicians with a critical reference that was helpful in evaluating the therapeutic effects and optimizing the clinical strategies. However, ^18^F-FDG PET/CT costs much and is too hard to maintain. Especially for majority of AE patients from poorly resourced areas, it is not readily available. In this background, our previous study pointed out that plasma IL-23 combined with IL-5 could be a marker indicating lesion metabolic activity with similar prognostic values as good as ^18^F-FDG PET/CT in patients with hepatic AE [7]. Plasma IL-23 measurement is a minimally invasive, accessible, and affordable technique especially for poverty. In the current study, we aim to explore the potential influence of albendazole administration on the circulating and regional immune profile in the experimental *E. multilocularis* infection model and key circulatory markers, if any, that are critical to albendazole administration treatment.

## 2. Materials and Methods

### 2.1. Ethics

This study was run under the frame of the *Guide for the Care and Use of Laboratory Animals* and the Institutional Animal Use and Care Committee of Xinjiang Medical University (approval number: 2017-123).

### 2.2. Animal

Six- to eight-week-old female BALB/c mice were purchased from the experimental animal center of Xinjiang Medical University. All mice were kept in a temperature-controlled, light-cycle room in animal facilities under specific pathogen-free conditions according to the national guidelines for animal care and use, with food and water *ad libitum*.

### 2.3. Maintenance and Isolation of *E. multilocularis* Metacestodes

Metacestodes were maintained by serial transplantation passages through i.p. injection in gerbil (*Meriones unguiculatus*). Four to ten weeks after injection, parasitic materials were procured under aseptic conditions. After washing with Hanks balanced salt solution (HBSS), the parasitic tissue was then grinded through a sterile 50 ml sieve, and acephalic vesicular cysts were suspended in 100 *μ*l RPMI-1640 (approximately 1000-1500 protoscolices per mice) in the preparation of injection.

### 2.4. Establishment of Murine Model

Laparotomy was performed after successful ether anesthesia, and protoscolices were injected into the left posterior lobe of the liver with a 1 ml syringe. Each experimental group included six animals unless otherwise stated. The control mice (mock infection) received intrahepatic injection of 100 *μ*l of RPMI-1640 into the same lobe. B-ultrasound was used to assess any potential parasitic lesion 1 and 2 months after the inoculation.

### 2.5. Albendazole Administration

Albendazole suspensions were prepared in carboxymethyl cellulose (CMC) 0.5% (*w*/*v*) in distilled water. Drug suspensions were freshly prepared each week and stored at -20°C for a period of seven days maximum. The control suspensions containing only CMC were treated identically. Treatments were initiated twelve weeks after infection and were repeated daily for 35 consecutive days.

### 2.6. Experimental Group

All animals were divided into four groups with six mice per group: (1) *E*. *multilocularis* infection group with albendazole treatment (*E.m*+ABZ), (2) *E. multilocularis* infection group without albendazole (*E.m*+CMC), (3) mice with albendazole administration (control+ABZ), and (4) mice without albendazole administration (control+CMC). Mice were sacrificed on the 36^th^ day after treatment, and specimens were collected.

### 2.7. Cell Sample Preparation

Under inhalational isoflurane (2%) anesthesia, the blood samples were obtained from the inferior vena cava. The prepared plasma was kept at -20°C until use. Spleen cell suspensions were prepared and depleted of erythrocytes by treatment with 0.83% NH_4_Cl in 0.01 m Tris–HCl (pH 7.2) and subsequently resuspended in RPMI-1640. Splenic cell suspensions were aliquoted into tubes and washed once in phosphate-buffered saline (PBS) for further flow cytometry and mRNA relative expression analysis.

### 2.8. Liver Tissue Preparation

The liver was procured, and both normal liver and AE lesions were sent for histopathology and PCR analysis. The specimens were sectioned for lesion, paralesion, and normal liver tissues and then reviewed by two independent senior pathologists.

### 2.9. Luminex Assay

Plasma concentrations of related cytokines and chemokines including IL-9 (sensitivity is 0.28 pg/ml), IL-12 (0.21 pg/ml), IL-17F (0.1 pg/ml), IL-22 (8.2 pg/ml), IL-23 (0.9 pg/ml), IL-27 (5.1 pg/ml), eotaxin (0.01 pg/ml), CCL2 (0.6 pg/ml), MIP-1a (1.1 pg/ml), MIP-1b (4.7 pg/ml), RANTES (0.2 pg/ml), GRO-*α* (2.8 pg/ml), CXCL8 (1.2 pg/ml), IP-10 (0.3 pg/ml), CXCL12*α* (20.5 pg/ml), MCP-1 (3.43 pg/ml), and MCP-3 (0.15 pg/ml) were detected using a Luminex bead-based multiplex assay (eBioscience) under close compliance with the manufacturer's guidelines. The brief procedure was as follows: Plasma samples were diluted using universal assay buffer first to meet the recommended analyte concentration. The beads were diluted and vortexed for 30 seconds, and 50 *μ*l of beads was added to each well of the plate. The plates were washed, and 25 *μ*l of samples was added to wells and was incubated with shaking for 60-120 min at room temperature (RT). After washing twice, we added 25 *μ*l of detecting antibody and incubated with shaking about 30 min at RT. We washed it twice followed by adding 50 *μ*l of streptavidin-PE and incubated with shaking for 30 min at RT. We washed it twice and resuspended using 120 *μ*l of reading buffer before acquiring data. Samples undetectable within the range of the assay were plotted as the detection limit. All Luminex measurements in a fluorescent signal were converted into concentrations in pg/ml.

### 2.10. RNA Isolation

Total RNA extraction was performed according to the instructions provided by the kit. 200 *μ*l chloroform was added to the appropriate mass of samples and then centrifuged for 15 min at 12000 rpm, 4°C, and then, 500 *μ*l isopropanol was added. Samples were again homogenized and centrifuged under the same conditions mentioned above. The supernatant was discarded, and the precipitate was washed in 1 ml 75% ethanol followed by centrifugation again under the same conditions, and after discarding the supernatant, the pellet was dried for about 15 min and then resuspended in RNAse-free ultrapure water. RNA concentration and quantity were estimated from the optical density at 260 and 280 nm, and RNA purity was estimated using 260/280 ratio 1.8 to 2.0. Total RNA was converted to cDNA using Revert Aid Reverse Transcriptase (Thermo Scientific), and oligo(dT)18 primers were stored at -20°C until analysis [8].

### 2.11. Real-Time Fluorescent Quantitative Reverse-Transcription Polymerase Chain Reaction (qRT-PCR)

The real-time PCR was conducted with the SYBR Green PCR premix following the manufacturer's protocols. The primers were purchased from Sangon, Shanghai, China. The data were performed using the SYBR Green program on i-Q5.0 Real-time PCR system (Bio-Rad, Foster City, CA, USA). The relative amounts of PCR products were determined using the relative standard curve method, and GAPDH was used as an internal control. The primer sequences are listed in Table 1. The 2^-*ΔΔ*Ct^ method was used to determine the specific Ct value of each target gene.

### 2.12. Immunohistochemistry

Liver tissue samples were prepared for immunohistochemistry analysis described as previously [9]. After heating at 60°C for approximately one hour, the slices were deparaffinized in xylene and rehydrated through graded alcohol. Citrate buffers were applied to retrieve the antigens, followed by quenching endogenous peroxidase activity with hydrogen peroxide. The samples were incubated overnight at 4°C with anti-mouse primary antibodies (Sino Biological Inc., China) at concentrations of 1 : 2000 and 1 : 400. A biotinylated secondary antibody labelled with streptavidin-horseradish peroxidase (ZSGB Biotech, Beijing, China) was applied to detect the antibodies through a DAB staining kit (ZSGB Biotech, Beijing, China). For the negative control, the primary antigens were replaced by PBS solutions.

### 2.13. Cell Preparation and Flow Cytometry Analysis

For analysis of Th17 cells, the splenic cell suspension was stimulated with 20 ng/ml phorbol 12-myristate-13-acetate and 1 *μ*g/ml ionomycin in the presence of 2 mmol/ml monensin (Sigma-Aldrich, St. Louis, Missouri, USA) in 24-well plates. After 6-hour culturing (37°C, 5% CO_2_), the cells were transferred to tubes and washed twice in PBS and centrifuged at 1500 rpm/min for 5 min; then, the supernatant was discarded. The cells were then incubated with fluorescein isothiocyanate (FITC) anti-human/mice CD4 monoclonal antibody (Ab) at 4°C in the dark for 30 min.

For the analysis of Treg cells, cells were incubated with anti-CD4-PERCP and anti-CD25-PE-CY7 for 30 min in the dark. Then, cells were washed with PBS twice and centrifuged at 1500 rpm/min for 5 min. The supernatant was discarded. After surface staining, the cells were fixed and permeabilized and then stained with anti-human/mice PE-IL-17/FoxP3 or isotype control. For flow cytometry analysis, cells were resuspended in PBS. Data were analyzed by FlowJo software.

### 2.14. Statistical Analysis

Statistical analysis was performed using the Statistical Package for the Social Sciences (SPSS), version 17.0. All continuous variables were expressed as median (interquartile range (IQR)) or number (%) in the text and tables. The Mann-Whitney *U* test was performed to detect the differences among groups. Spearman correlation analysis was used as a test of correlation between two continuous variables and was determined by Spearman correlation coefficients. All tests were two sided, and a value of *p* ≤ 0.05 was considered statistically significant.

## 3. Results

### 3.1. Success Rate of Animal Model

**Figure 1:**
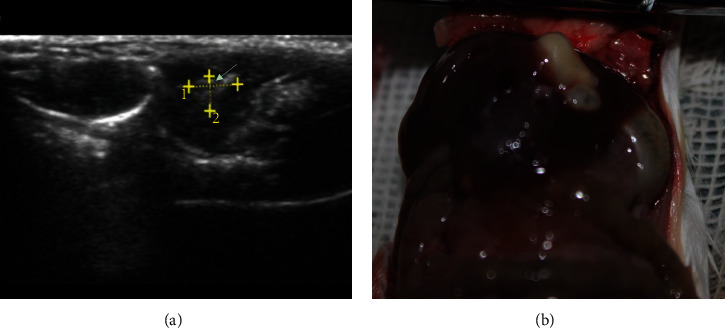
Intrahepatic AE lesion assessment. (a) Ultrasound shows a hyperechoic lesion in the liver. (b) Laparotomy shows whitish liver lesion in the left lobe.

The presence of a hepatic AE lesion was confirmed by ultrasound at designated timepoints of 1 and 2 months and finally confirmed by laparotomy. The total success rate of intrahepatic infection was 70% (Figure 1).

### 3.2. Plasma Concentration of Cytokines and Chemokines

T cell subsets including Th1, Th2, Th9, Th17, and Treg cells were actively involved during the development of *E. multilocularis* infection. Herein, related six cytokines and eight chemokines were studied, and data are shown in Table 2.

#### 3.2.1. The Concentrations of Cytokines in Different Groups

**Figure 2:**
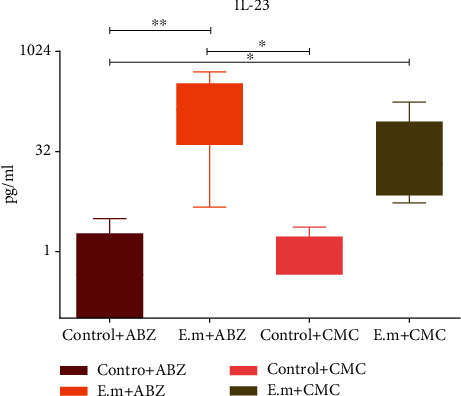
The plasma level of IL-23 detected by ELISA. The level of IL-23 in the AE-infected groups was higher than that in the control groups, and it further increased after albendazole administration. Abbreviations: ABZ: albendazole; CMC: carboxymethyl cellulose; IL: interleukin. ^∗^*p* < 0.05, ^∗∗^*p* < 0.01.

The plasma levels of IL-9, IL-12, IL-17F, and IL-27 were slightly higher in *E. multilocularis*-infected mice without albendazole treatment compared to those with albendazole albeit without statistical significance (*p* > 0.05). Conversely, the plasma level of IL-22 was lower in *E. multilocularis*-infected mice without albendazole treatment than those with albendazole treatment (*p* > 0.05). Plasma levels of IL-23 were significantly higher in *E. multilocularis*-infected mice when compared with the control group; furthermore, more obvious increasing levels were found after albendazole treatment (*p* < 0.05; Figure 2).

#### 3.2.2. The Concentrations of Chemokines in Different Groups

**Figure 3:**
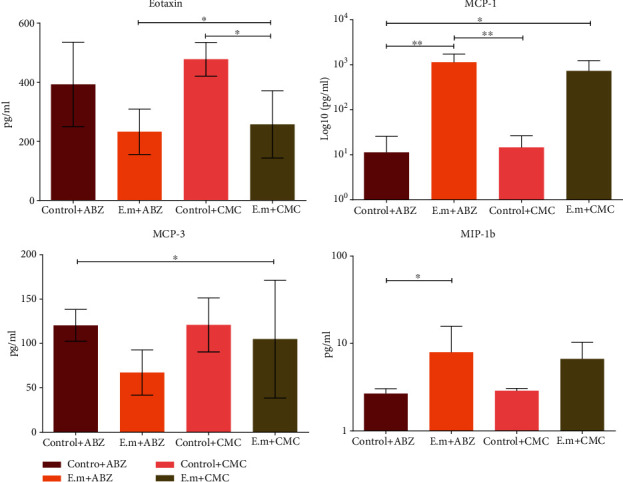
Plasma concentration of chemokines in different groups. The plasma level of eotaxin and MCP-3 in the AE-infected groups was lower than that in the control groups while the plasma level of MCP-1 and MIP-1b performed adversely. Abbreviations: ABZ: albendazole; CMC: carboxymethyl cellulose; MCP: monocyte chemoattractant protein; MIP: macrophage inflammatory protein. ^∗^*p* < 0.05, ^∗∗^*p* < 0.01.

Eight chemokines related to different T cell subsets were analyzed. The plasma levels of all chemokines other than eotaxin, MCP-1, and MCP-3 were slightly higher in *E. multilocularis*-infected mice compared to the control group (*p* > 0.05, *p* < 0.05, and *p* > 0.05). Plasma levels of eotaxin were significantly decreased in *E. multilocularis*-infected mice with albendazole treatment (*p* < 0.05). Besides, levels of GRO-*α*, MCP-3, MIP-1*α*, and RANTES were slightly lower in *E. multilocularis*-infected mice with albendazole treatment compared to single *E. multilocularis*-infected without treatment (*p* > 0.05). Conversely, levels of IP-10 and MIP-1*β* showed an increased tendency in *E. multilocularis*-infected mice with albendazole compared to those without treatment (*p* > 0.05; Figure 3).

### 3.3. Real-Time PCR Analysis

**Figure 4:**
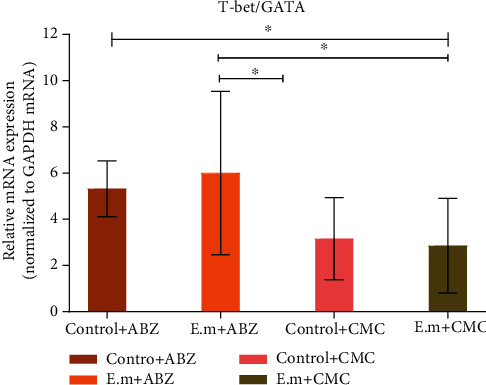
Related cytokines and transcription factors detected by RT-PCR, which were statistically significant. The relative mRNA expression levels of T-bet and the ratio of T-bet/GATA-3 in the AE-infected group were decreased than that in the control group while the level of TLR2 and TLR4 in the AE-infected group was increased compared to that in the control group. Abbreviations: ABZ: albendazole; CMC: carboxymethyl cellulose; TLR: Toll-like receptor. ^∗^*p* < 0.05.

Th1/Th2/Th17/Treg-related cytokines and their specific transcription factors and functional cytokines were detected in splenic cell suspension by qRT-PCR. The detailed expression levels of all cytokines and transcription factors are tabulated in Table 3. The expression levels of INF-*γ*, IL-2, IL-10, and IL-17A have shown no statistical significance between the groups. Th1 cell-specific transcription factor and its ratio to GATA3 were significantly higher in *E. multilocularis*-infected mice with albendazole administration. No statistical significance was found regarding the expression levels of other cytokines and transcription factors (*p* > 0.05). Moreover, the expression levels of both TLR2 and TLR4 expression levels were higher in the *E. multilocularis*-infected mice compared to the control group, and more noteworthily, their levels were decreased with albendazole administration (Figure 4). As shown in Table 4, no statistical difference was found among the lesion, para-lesion, and normal groups. And normal tissue in the four groups was the same.

### 3.4. Immunohistochemical Analysis

**Figure 5:**
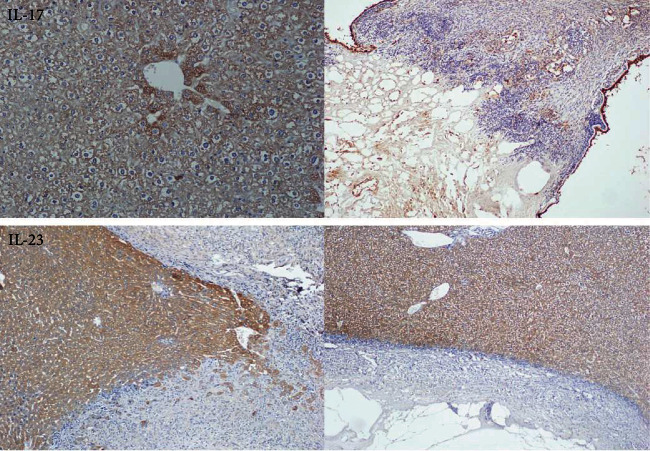
The expression of the IL-17 and IL-23 antigens in the AE liver tissue after albendazole administration.

We determined the expression and distribution of IL-17A and IL-23 in liver tissues by immunohistochemistry. Representative immunohistochemical results are shown in Figure 5.

### 3.5. Levels of T Cell Subpopulations in splenic cell suspension

**Figure 6:**
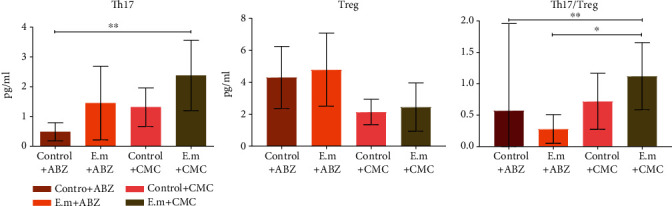
CD4^+^ IL-17^+^ Th17 cells and CD4^+^ CD25^+^ FoxP3^+^ Treg cells in splenic cell suspension. The level of Th17 and Th17/Treg ratio in the AE-infected group were decreased after albendazole administration. ABZ: albendazole; CMC: carboxymethyl cellulose; Th cell: T helper cell; Treg cell: regulatory T cell.

We have detected T cell subpopulations including CD4^+^ IL-17^+^ Th17 cells and CD4^+^ CD25^+^ FoxP3^+^ Treg cells in splenic cell suspension. The frequency of the CD4^+^ IL-17^+^ Th17 cell was higher in *E. multilocularis*-infected mice compared to the control group. Meanwhile, it was higher in mice without albendazole administration in both normal and *E. multilocularis*-infected mice, albeit with no statistical significance (*p* > 0.05). The frequency of CD4^+^ IL-17^+^ Th17 cells was significantly higher in the *E. multilocularis*+CMC group compared to the control+ABZ group (*p* < 0.05). The CD4^+^ CD25^+^ FoxP3^+^ Treg cells show a reciprocal role in immune response; its frequency was higher in *E. multilocularis*-infected mice compared to the normal control. Meanwhile, it was higher in mice with albendazole administration in both normal and *E. multilocularis*-infected mice, albeit with no statistical significance (*p*>0.05). The ratio of Th17 to Treg cells was calculated, and it was higher in *E. multilocularis*-infected mice compared to the control, albeit with no statistical significance (*p* > 0.05). However, the ratio was markedly decreased with albendazole administration in both groups (Figure 6).

## 4. Discussion

This study presented a comprehensive analysis of different T cell subsets including Th1/Th2/Th9/Th17/Treg, related cytokines, chemokines, and transcription factors in mice inoculated with *E. multilocularis* and their alteration under albendazole administration. To the best of our knowledge, this is the first comprehensive experimental survey with regard to albendazole with related immune parameters, opening pathways for future intervention and mechanism.

After successful ingestion into the alimentary tract and subsequently into the portal vein, the oncosphere of *E. multilocularis* resides itself in the host's liver and therefore forms an infiltrative mass [10]. It is widely accepted that the essence of AE lesion formation is represented by the crosstalk between the parasite and the host's immune system. The critical role of T helper cells was proven with a specific dominant type during different special stages of the infection. As involved in the proinflammatory profile, Th1 and Th17 cell subsets are related to the clearance of the parasite, while Th2 and Treg cells favor the maintenance of the parasite with their anti-inflammatory features [10–13]. At the initial stage of infection, the host's immune system severely responds against the parasite, and therefore, a strong Th1- and Th17-type immune response was dominant; conversely, through the development, the parasite is able to modulate the immune system providing immune tolerance with Th2- and Treg-dominant immune response type [12]. The immune response may differ according to the site and stage of infection. Herein, we have developed a hepatic *E. multilocularis* infection model in BALB/c mice to simulate the clinical AE infection. Although this is not a primary infection, however, this model is easy to practice with good reproducibility and, to some extent, mimics the clinical scenario.

Similar to our previous clinical finding, Th17 cell frequency, Th17/Treg ratio, IL-17, and IL-23 showed circulatory and/or regional expression [7]. In addition, such a change could be attributed to the host's enhanced immune response to the parasite and the local inflammatory reaction in the liver. Furthermore, the increased levels of TLR2 and TLR4 could be related to the immune tolerance process induced by the parasite which was also supported by our previous reports [8, 14]. Studies have shown that Th17/Treg imbalance plays an important role in the immune interaction between the host and echinococcosis [15]. The Th17 cell is a new type of T lymphocyte subset that distinguishes Th1 and Th2 effector T lymphocytes; the specific transcription factor ROR*γτ*, IL-17, IL-23, and TNF-*α* play a key role in the occurrence and development of parasites [16]. The transcription factor of Treg cells and FoxP3 plays an anti-inflammatory role by mediating the release of IL-10 and TGF-*β*1 and maintaining tolerance to its own or foreign components [17]. Interestingly, ROR*γτ* can inhibit the differentiation of Treg cells while FoxP3 can do the same to Th17, which reflects that they antagonize each other and maintain balance in immunity [18]. It is quite worthy to note that the paralesion area is quite pivotal for both immune response and clinical decision-making process. In the current study and our previous work on resected AE lesions, we have found highly expressed IL-17 and IL-23 in response to the parasite in the liver parenchyma [7, 15]. From the point of immune view, Th1/T2 and Th17/Treg along with other immune cells decide the destiny of the parasite from the progressive AE lesion to the abortive lesion [13, 19]. Meanwhile, from the point of clinical view, the activity of this region directly influences the period of albendazole administration timing, discontinuation, and even surgical resection margin.

Chemokines including eotaxin are actively involved in the promotion of eosinophil accumulation in the lesion to limit the infection, and therefore, the altitude of the eotaxin level could reflect the severity of tissue infection [20, 21]. Besides, eotaxin has been proved to be important in the clearance of parasite, and parasites reduce inflammatory response in the way of decreasing the recruitment of eosinophils in order to facilitate the existence within the host [22, 23]. Similar with previous findings, the level of eotaxin was increased in *E. multilocularis*-infected mice but decreased after albendazole administration.

Previously, we have indicated that IL-23 combined with IL-5 could be a surrogate marker for metabolic activity [7]. In the current experimental work, IL-23 was markedly increased in *E. multilocularis*-infected mice, and further elevation was observed with albendazole administration. Such an alteration might be attributed to enhanced parasite clearance by albendazole itself and potential harsh hepatic regional response.

Albendazole is effective against parasites, but the specific time of administration is not clear, which brings unnecessary harm to patients. Experts agree that patients with AE still need to take BMZ for at least two years and should be followed up for ten years after radical resection [4]. The disappearance of *anti-Em18* antibodies, calcification of more than 50% of the primary lesions, and negative PET-CT scan can be used as indications for drug withdrawal [4, 24]. Expensive follow-up costs and scarce medical resources make it hard and even impossible for most patients to complete. In our previous human study, we have extensively focused on the potential marker that may distinct the metabolic active AE from metabolically inactive lesion, and the results demonstrated that plasma levels of IL-23 and IL-5 could be used as a good marker [7]. In the present study, we have first comprehensively examined both circulating and regional immune profile in mice with intrahepatic *E. multilocularis* infection and found that IL-23 was markedly higher in infected mice with albendazole administration indicating that after having been treated with albendazole, the parasite showed a lower immunosuppression reaction; thus, there was a high predominance of Th17 cell rather than Treg cells.

## 5. Conclusion

Albendazole administration influenced the balance of immune response and promoted the secretion of proinflammatory factors which is beneficial to parasite clearance. IL-23 seems to be associated with the successful albendazole treatment in mice with *E. multilocularis* infection; such a change could be translated into clinical application in the near future.

## Figures and Tables

**Table 1:** Primers used for real-time PCR analysis.

Gene	Forward primer (5′-3′)	Reverse primer (5′-3′)	Fragment length (bp)	NCBI reference sequence
IL-1*β*	AAGGGCTGCTTCCAAACCTTTGAC	ATACTGCCTGCCTGAAGCTCTTGT	100	XM_006498795.5
IL-2	AGCAGGATGGAGAATTACAGGAAC	AATCCAGAACATGCCGCAGA	139	NM_008366.3
IL-4	TCAACCCCCAGCTAGTTGTC	AAATATGCGAAGCACCTTGG	227	NM_021283.2
IL-10	TGCACTACCAAAGCCACAAG	TAAGAGCAGGCAGCATAGCAG	84	XM_036162094.1
IL-17A	ATCCCTCAAAGCTCAGCGTGTC	GGGTCTTCATTGCGGTGGAGAG	170	NM_010552.3
IL-23	CCAGCGGGACATATGAATCTACT	CTTGTGGGTCACAACCATCTTC	97	NM_031252.2
IFN-*γ*	ACTGGCAAAAGGATGGTGAC	GACCTGTGGGTTGTTGACCT	212	XM_021175069.1
ROR*γτ*	GAACCAGAACAGGGTCCAGA	TCGGAAGGACTTGCAGACAT	120	XM_006501163.4
Foxp3	CACCTATGCCACCCTTATCCG	CATGCGAGTAAACCAATGGTAGA	91	XM_021152692.2
GATA3	GGCACGATCCAGCACAGAA	TGGTAGAGTCCGCAGGCAT	114	XM_030247533.2
T-bet	CCTCTTCTATCCAACCAGTATCCTG	TTCATAACTGTGTTCCCGAGGTG	120	XM_021177762.1
TLR2	ACAGCTACTGTGTGACTCTCCGCC	GGTCTTGGTGTTCATTATCTTGCGC	601	XM_030252611.2
TLR4	CAGAGTTTCCTGCAATGGATCA	GCTTATCTGAAGGTGTTGCACAT	85	NM_021297.3
TGF-*β*	CTTCAGCTCCACGGAGAAGA	AGCGCCTGCGGCACGCAGCA	228	XM_021167684.1
GAPDH	TGCACCACCAACTGCTTAG	GGATGCAGGGATGATGTTC	177	XM_036165840.1

**Table 2:** Plasma concentrations of cytokines and chemokines in different groups (pg/ml).

Item/group	Control+ABZ	Control +CMC	*E.m*+CMC	*E.m*+ABZ	*p* value
Cytokines					
IL-9	5.165 ± 3.4151	7.806667 ± 12.76623	13.86 ± 11.11818	6.628333 ± 3.49739	>0.05
IL-12	44.44543 ± 22.16263	42.202 ± 33.51094	20.9815 ± 14.80863	19.55888 ± 15.4455	>0.05
IL-17F	64.18418 ± 35.42619	38.80851 ± 8.971003	91.2388 ± 83.92481	30.62029 ± 30.77487	>0.05
IL-22	90.69833 ± 56.37968	77.595 ± 113.4873	108.765 ± 79.6351	154.5633 ± 107.8698	>0.05
IL-23	0.916667 ± 1.212809^*Δ*,♠^	1.19 ± 0.718415^♦^	48.81333 ± 66.3169^♠^	186.575 ± 188.6204^♦,*Δ*^	<0.05
IL-27	12.22667 ± 15.33405	13.05667 ± 26.97575	18.052 ± 16.15805	15.46333 ± 18.28892	>0.05
Chemokines					
Eotaxin	392.7833 ± 392.7833	477.7183 ± 57.06299^∗^	257.355 ± 113.9356^∗^^,#^	232.3867 ± 76.68778^#^	<0.05
GRO-a	10.285 ± 10.90091	9.173333 ± 5.435423	29.758 ± 29.95188	51.23667 ± 72.28047	>0.05
IP-10	22.30667 ± 7.106275	29.165 ± 6.060095	31.27833 ± 24.85722	19.43333 ± 7.437383	>0.05
MCP-1	11.34667 ± 14.39924*^Δ^*	14.64333 ± 11.8182^♦^	730.6517 ± 505.2256	1141.032 ± 585.3943^*Δ*,♦^	<0.05
MCP-3	120.4083 ± 17.86446	120.9983 ± 30.45888	104.9233 ± 66.38534	67.27 ± 25.42277	>0.05
MIP-1a	2.673333 ± 1.110489	4.083333 ± 4.449219	19.16333 ± 30.78809	5.016667 ± 2.466306	>0.05
MIP-1b	2.678333 ± 0.355889^♠^	2.888333 ± 0.179155	6.65 ± 3.698032^♠^	7.941667 ± 7.793583	>0.05
RANTES	43.07667 ± 16.01221	56.49167 ± 20.55444	64.47167 ± 57.81003	43.84 ± 14.671	>0.05

^∗^The control+CMC group vs. AE+CMC group; ^#^the AE+CMC group vs. AE+ABZ group; *^Δ^*the control+ABZ group vs. AE+ABZ group; ^♦^the control+CMC group vs. AE+ABZ group; ^♠^the control+ABZ group vs. AE+CMC group. Abbreviations: ABZ: albendazole; CMC: carboxymethyl cellulose; AE: alveolar echinococcosis; IL: interleukin; GRO: growth-related oncogene; IP: interferon- (IFN-) *γ*-induced protein; MCP: monocyte chemoattractant protein; MIP: macrophage inflammatory protein.

**Table 3:** Relative mRNA expressions of specific transcription factors and functional cytokines of T cell subtypes in splenic cell suspension.

Item/group	Control+ABZ	AE+ABZ	Control +CMC	AE+CMC	*p* value
INF-*γ*	0.14821832 ± 0.050183528	0.1880775 ± 0.185361291	0.4323275 ± 0.468856209	0.5654325 ± 0.593368424	>0.05
IL-2	0.056276 ± 0.032162	0.093628 ± 0.165066	0.18693 ± 0.269571	0.084669 ± 0.106371	>0.05
IL-4	0.005645 ± 0.004307	0.012071 ± 0.01043	0.01427 ± 0.013435	0.033663 ± 0.072105	>0.05
IL-10	0.000352 ± 0.000244	0.000221 ± 0.000249	0.000578 ± 0.000514	0.000866 ± 0.000815	>0.05
IL-17A	0.000 ± 0.000	0.000 ± 0.000	0.000012 ± 0.000009.39	0.00001.14 ± 0.0000174	>0.05
T-bet	0.093657 ± 0.018695^♠*Δ*^	0.088425 ± 0.038359	0.057264 ± 0.03253^♠^	0.057395 ± 0.031037*^Δ^*	<0.05
GATA3	0.017773 ± 0.002158	0.017828 ± 0.010741	0.02012 ± 0.01079	0.024986 ± 0.013217	>0.05
T-bet/GATA3	5.320536 ± 1.207308*^Δ^*	6.001664 ± 3.528325^∗^^♦^	3.160419 ± 1.776575^∗^	2.852609 ± 2.053544^*Δ*♦^	<0.05
ROR*γτ*	0.000313 ± 0.000142	0.000548 ± 0.000319	0.000371 ± 0.000262	0.000345 ± 0.000268	>0.05
FoxP3	0.0000725 ± 0.00002.66	0.000065 ± 0.0000444	0.0000325 ± 0.0000271241	0.00006375 ± 0.00005344	>0.05
ROR*γτ*/FoxP3	4.31475885 ± 1.122122639	9.75488445 ± .207780231	22.88125 ± 8.5720635	8.84583333 ± 9.542202625	>0.05
TLR2	0.009476 ± 0.008194192	0.00479 ± 0.004621002	0.0093275 ± 0.012912928	0.018155 ± 0.013105589	<0.05
TLR4	0.00178 ± 0.000803	0.0029475 ± 0.001363093	0.00229375 ± 0.001369045	0.0046575 ± 0.004788993	<0.05

^∗^The control+CMC group vs. AE+CMC group; *^Δ^*the control+ABZ group vs. AE+ABZ group; ^♠^the control+ABZ group vs. AE+CMC group. Abbreviations: ABZ: albendazole; CMC: carboxymethyl cellulose; AE: alveolar echinococcosis; IL: interleukin; GRO: growth-related oncogene; IP: interferon- (IFN-) *γ*-induced protein; MCP: monocyte chemoattractant protein; MIP: macrophage inflammatory protein.

**Table 4:** mRNA expression of cytokines and chemokines in each sampling liver tissue.

Group/item		TGF-*β*	IL-23	IL-1b	TNF-*α*	FoxP3
Lesion	E.m+CMC	1.462 ± 0.090	1.777 ± 0.250	1.846 ± 0.240	1.790 ± 0.088	1.739 ± 0.229
	E.m+ABZ	1.424 ± 0.107	1.770 ± 0.186	1.691 ± 0.332	1.649 ± 0.201	1.541 ± 0.294

Paralesion	E.m+CMC	1.489 ± 0.155	1.803 ± 0.113	1.809 ± 0.231	1.801 ± 0.270	1.729 ± 0.233
	E.m+ABZ	1.494 ± 0.029	1.806 ± 0.092	1.872 ± 0.239	1.807 ± 0.170	1.615 ± 0.301

Normal	E.m+CMC	1.427 ± 0.127	1.784 ± 0.102	1.759 ± 0.243	1.709 ± 0.249	1.645 ± 0.332
	E.m+ABZ	1.429 ± 0.090	1.784 ± 0.165	1.698 ± 0.105	1.870 ± 0.231	1.796 ± 0.317

*p* value		>0.05	>0.05	>0.05	>0.05	>0.05

Normal	E.m+CMC	1.427 ± 0.127	1.784 ± 0.102	1.759 ± 0.243	1.709 ± 0.249	1.645 ± 0.332
	E.m+ABZ	1.429 ± 0.090	1.784 ± 0.165	1.698 ± 0.105	1.870 ± 0.231	1.796 ± 0.317
	Control+CMC	1.424 ± 0.100	1.888 ± 0.125	1.722 ± 0.119	1.818 ± 0.095	1.785 ± 0.115
	Control+ABZ	1.363 ± 0.195	1.820 ± 0.250	1.658 ± 0.212	1.719 ± 0.218	1.620 ± 0.224

*p* value		>0.05	>0.05	>0.05	>0.05	>0.05

ABZ: albendazole; CMC: carboxymethyl cellulose; IL: interleukin; TGF-*β*: transforming growth factor-*β*; TNF-*α*: tumor necrosis factor-*α*; FoxP3: forkhead box P3.

## Data Availability

The data used to support the findings of this study are available from the corresponding authors upon reasonable request.
